# Supplementary material for: A high-performance speech neuroprosthesis
Source: Nature. 2023 Aug 23;620(7976):1031–6. doi: 10.1038/s41586-023-06377-x (PMC10468393; doi:10.1038/s41586-023-06377-x)
Supplement: Supplementary file 1 — This file contains Supplementary methods. [file 41586_2023_6377_MOESM1_ESM.pdf]

nature portfolio

https://doi.org/10.1038/s41586-023-06377-x

Supplementary information

A high-performance speech neuroprosthesis

In the format provided by the authors and unedited

Contents

1 Experimental procedures 2
1.1 Study participant . . . 2
1.2 Functional MRI speech lateralization . . . 2
1.3 Array placement targeting . . . 3
1.4 Neural signal processing . . . 3
1.5 Data collection rig . . . 4
1.6 Overview of data collection sessions . . . 4
1.7 Instructed delay tasks . . . 8
1.8 Voiced vs. silent speaking behavior . . . 8
1.9 Decoder evaluation sessions . . . 9
1.10 Sentence selection . . . 9
2 Neural representation of orofacial movements and speech in orofacial cortex 10
2.1 Tuning heat maps . . . 10
2.2 Naive Bayes classification . . . 10
2.3 Preserved articulatory representation of phonemes . . . 11
2.3.1 Electromagnetic articulography (EMA) representations . . . 11
2.3.2 Saliency vector representation . . . 11
2.3.3 Similarity matrices . . . 12
2.3.4 Correlation between articulatory and neural representations . . . 12
2.3.5 Low-dimensional visualization of phoneme geometry . . . 12
2.3.6 Place and formant representation . . . 12
2.3.7 Decoder labeling representation . . . 13
2.3.8 Single phoneme task representation . . . 13
2.4 Neural correlation across days . . . 13
3 Phoneme transcription and labelling 14
4 Decoder performance metrics 14
4.1 Word and phoneme error rates . . . 14
4.2 Words per minute . . . 14
5 RNN architecture 15
5.1 Feature pre-processing and day-specific input layers . . . 16
5.2 Rolling z-scoring . . . 16
6 RNN training overview 16
6.1 Connectionist temporal classification (CTC) loss . . . 16
6.2 Artificial noise . . . 17
6.3 Supervised training . . . 17
7 Offline performance sweeps 18
7.1 Overview . . . 18
7.2 Effect of channel count on performance . . . 18
7.3 Amount of training data . . . 18
7.4 Language model vocabulary size sweep . . . 18
8 Language model 19
8.1 Overview . . . 19
8.2 OpenWebText2 preprocessing . . . 19
8.3 Constructing the n-gram language model . . . 19
8.4 Inference with the n-gram language model . . . 20
8.5 Offline language model optimization . . . 20
9 Statistics 21

1. Experimental procedures

1.1. Study participant

This study includes data from one participant (identified as T12) who gave informed consent and was enrolled in the BrainGate2 Neural Interface System clinical trial (ClinicalTrials.gov Identifier: NCT00912041, registered June 3, 2009). This pilot clinical trial was approved under an Investigational Device Exemption (IDE) by the US Food and Drug Administration (Investigational Device Exemption #G090003). Permission was also granted by the Institutional Review Board of Stanford University (protocol #52060). T12 gave consent to publish photographs and videos containing her likeness. All research was performed in accordance with relevant guidelines/ regulations.

T12 is a left-handed woman, 67 years old at the time of data collection, with slowly-progressive bulbar-onset Amyotrophic Lateral Sclerosis (ALS) diagnosed at age 59 (ALS-FRS score of 26 at the time of study enrollment). On March 30, 2022, four 64-channel, 1.5 mm-length silicon micro electrode arrays coated with sputtered iridium oxide (Blackrock Microsystems, Salt lake City, UT) were implanted in T12's left hemisphere, based on preoperative anatomical and functional magnetic resonance imaging (MRI) and cortical parcellation (see sections 1.2 and 1.3 below for details). Two arrays were placed in area 6v (oral-facial motor cortex) of ventral precentral gyrus, and two were placed in area 44 of inferior frontal gyrus (considered part of Broca's area). Data are reported from post-implant days 27-148. On average, 119.6 ± 5.0 (Mn ± sd) out of 128 electrodes recorded spike waveforms at a rate of at least 2 Hz when using a spike-detection threshold of -4.5 RMS, where RMS is the electrode-specific root mean square of the voltage time series recored on that electrode (see Extended Data Fig. 1 for example waveforms).

T12 is severely dysarthric due to bulbar ALS and has been for nearly 8 years. She retains partial use of her limbs, and communicates primarily through use of a writing board or iPad tablet. She is able to vocalize while attempting to speak, and is able to produce some subjectively differentiable vowels sounds. However, we had difficulty discerning nearly all consonants produced in isolation (with the possible exception of the bilabial nasal consonant "M"), and could not reliably make out any consonants or vowels when T12 attempted to speak whole sentences at a fluent rate (SVideo 1 shows examples of attempted speaking).

1.2. Functional MRI speech lateralization

Prior to surgery, participant T12 underwent anatomic and functional brain imaging on a GE Discovery MR750 3T MRI scanner, using a routine clinical acquisition protocol, in order to determine whether she was right or left hemisphere dominant for language. BOLD fMRI images were acquired using T2*-weighted volumes collected with 4 mm slice thickness and 2x2 mm^2 in-plane voxel resolution. BOLD images were acquired during performance of a suite of tasks including visually responsive naming, object naming, auditory responsive naming, repetitive movements of the right hand, left hand, right foot, left foot, and tongue. Tasks were performed in 4 minute blocks consisting of repeated sequences of 10 seconds of task performance followed by 10 seconds of rest. Task instructions were presented by the SensaVue presentation system with verbal instructions given by the MRI technologist. T-score thresholds for processing were chosen based on direct inspection of the preliminary fMRI output for each task produced by the scanner software and inspected by the interpreting radiologist at the scanner.

Following scan completion, the complete data set was sent to and processed by DynaSuite. Fully processed fMRI statistical parametric maps for each task were registered to and overlaid on an anatomic 3D gradient echo T1-weighted (BRAVO) image acquired with 1 mm isotropic voxels. Quality control steps including motion tracking and assessment of anatomic-functional registration fidelity. Language lateralization was assessed by visual inspection of lateralization of activation in Broca's area, Wernicke's area, speech supplemental motor area, and the basal temporal language area. Results indicated a clear left hemisphere lateralization of language in T12.

1.3. Array placement targeting

The surgical targets for array placement within areas 6v and 44 were selected based on gross anatomical structure (e.g., gyri and sulci), vasculature, and estimates of the boundaries of areas 44 and 6v obtained using a cortical parcellation method derived from multi-modal Human Connectome Project (HCP) data [39].

We acquired T1-weighted (T1w), T2-weighted (T2w), resting-state functional MRI (rsfMRI), single band fMRI, and spin echo fieldmap images to generate the HCP-based cortical parcellation. The participant was scanned in a 3T Ultra High Performance scanner (GE Healthcare) with a Nova 32-channel coil. Scan parameters were based on HCP Lifespan protocols and modified for the GE system (Table 1).

Data were processed using the HCP pipelines as described on https://github.com/Washington-University/HCPpipelines (see Glasser et al. 2016 for further details). Briefly, T1w and T2w images were initially preprocessed using the FreeSurfer pipeline (version 7.1.1) to perform motion, distortion, and bias field corrections; brain extraction; white matter segmentation; cortical surface reconstruction, and spherical mapping (PreFreeSurferPipelineBatch.sh, FreeSurferPipelineBatch.sh). Surface outputs were then aligned to the standard surface template using MSMSulc, as well as used to create myelin maps (PostFreeSurferPipelineBatch.sh).

rsfMRI data were corrected for motion, bias field, and susceptibility distortions using the spin echo fieldmaps and single band reference fMRI images and non-linearly registered to MNI space (GenericfMRIVolumeProcessingPipelineBatch.sh), followed by volume to surface mapping (GenericfMRISurfaceProcessingPipelineBatch.sh). Data then underwent spatial MELODIC ICA (IcaFixProcessingBatch.sh), manual classification of the components as signal or noise, and denoising.

The MSMAll pipeline was then run to re-align the participant's cortical surface to the standard surface template using areal features from the cortical folding map, myelin map, rsfMRI networks, and rsfMRI-based retinotopy. Lastly, the data also underwent a dedrift and resample step (DeDriftAndResamplePipelineBatch.sh). This then allowed us to overlay the Human Connectome Project (HCP) cortical parcellation (210P) and areal confidence (210V) maps [39] onto the participant's brain using Connectome Workbench, thereby obtaining estimates of areas 6v and 44 within the participant's brain (as shown in Extended Data Fig. 2). In Extended Data Fig. 2E-F we show resting state network 25 (an ICA component originally identified in [39] as the "Language RSN" shown there in supplemental figure 8a).

1.4. Neural signal processing

Neural signals were recorded from the microelectrode arrays using the Neuroplex-E system (Blackrock Microsystems) and transmitted via a cable attached to a percutaneous connector. Signals were analog filtered (4th order Butterworth with corners at 0.3 Hz to 7.5 kHz), digitized at 30 kHz (250 nV resolution), and fed to custom software written in Simulink (Mathworks) for digital filtering and feature extraction. Digital filtering began with a highpass filter (300 Hz cutoff) that was applied non-causally to each electrode, using a 4 ms delay, in order to improve spike detection [40]. Linear regression referencing (LRR) was then applied to further reduce reduce ambient noise artifacts [41].

After filtering, binned threshold crossing counts (20 ms bins) were computed by counting the number of times the filtered voltage time series crossed an amplitude threshold set at -4.5 times the standard deviation of the voltage signal. Electrode-specific thresholds and LRR filter coefficients were set using data recorded from an initial "diagnostic" block at the beginning of each session (see section 1.7 for more details). Binned spike band power (20 ms bins) was computed by taking the sum of squared voltages observed during each time bin. Threshold crossing rates and spike band power are commonly used measurements of local spiking activity that have been shown to be comparable to sorted single unit activity in terms of decoding performance and neural population structure [42, 43, 44]. For decoding, threshold crossing counts and spike band power from the 128 electrodes in area 6v were

Image	T1w	T2w	rsfMRI	rsfMRI-single band	Spin echo fieldmap
Sequence	3D MPRAGE	3D CUBE	2D Gradient Echo EPI	2D Gradient Echo EPI	2D Spin Echo EPI
TR (ms)	3000	2500	800	4200	8000
TE (ms)	3.5	60-78	37	30	min full
TI (ms) 1060	-	-	-	-	-
Parallel imaging	2 x 1.25	1.9 x 1.9	-	-	-
Fat suppression	no	no	yes	yes	yes
Resolution (mm)	0.8 x 0.8 x 0.8	0.8 x 0.8 x 0.8	2 x 2 x 2	2 x 2 x 2	2 x 2 x 2
Matrix size	320 x 320 x 230	320 x 320 x 216	104 x 104 x 72	104 x 104 x 72	104 x 104 x 72
FOV (mm)	256 x 256 x 184	256 x 256 x 184	208 x 208 x 144	208 x 208 x 144	208 x 208 x 144
Flip angle	8	-	54	90	-
Slice orientation	sagittal, AC-PC	sagittal, AC-PC	axial AC-PC	axial AC-PC	axial AC-PC
Phase encoding	-	-	AP and PA (separately)	AP and PA (separately)	AP and PA (separately)
Multiband factor	-	-	8	1	-

Table 1. *MRI Scan Parameters for Cortical Parcellation.*

concatenated to yield a 256 x 1 feature vector per time step. For neural tuning analyses (e.g. Figure 1), only threshold-crossing counts were used.

1.5. Data collection rig

Digital signal processing and feature extraction was performed on a dedicated computer using Simulink Real-Time. Extracted features were then sent to a separate computer running Ubuntu for neural decoding and recording. Decoding and recording software was written in Python using TensorFlow 2 and Redis. The Ubuntu computer also ran the experimental task software that displayed cues to T12 on a computer monitor. The task software was implemented using MATLAB and the Psychophysics Toolbox [45]). Finally, a third computer running Windows was used to interface with the Neuroplex-E system and control the starting and stopping of experimental tasks.

1.6. Overview of data collection sessions

Neural data were recorded in 2-4 hour “sessions” on scheduled days, which typically occurred 2 times per week. During the sessions, T12 sat in either a wheelchair or power lift chair in an upright position, with a pillow placed to support her head and neck, and her hands resting on her lap. A computer monitor placed in front of T12 indicated which sentence to speak (or which movement to make) and when. Data were collected in a series of 5-10 minute “blocks” consisting of an uninterrupted series of trials. In between these blocks, T12 was encouraged to rest as needed. Table 2 below lists all 27 data collection sessions reported in this work.

Table 2: Data Collection Sessions

Session Number	Date (Post-Implant Day)	Description	Data

1	2022.04.21 (22)	Phoneme and oro-facial movement sweeps	• 16 repetitions of each of the 39 English phonemes<br>• 20 repetitions each of various orofacial movements (tongue, lips, jaw, cheeks, larynx, forehead, eyelids)
2	2022.04.26 (27)	Phoneme sweep	• 20 repetitions of each of the 39 English phonemes
3	2022.04.28 (29)	Initial training data day #1	• 300 Open WebText training sentences
4	2022.05.03 (34)	Individual words from the 50-word set [46]	• 20 repetitions of each of 50 individual words from the Moses et al 2021 vocabulary [46]
5	2022.05.05 (36)	Initial training data day #2	• 380 Open WebText training sentences
6	2022.05.17 (48)	Initial training data day #3	• 440 Open WebText training sentences<br>• 50 Moses sentences used for offline assessment (not training)
7	2022.05.19 (50)	Initial training data day #4	• 200 Open WebText training sentences<br>• 50 Moses sentences used for offline assessment (not training)
8	2022.05.24 (55)	Real-time decoding pilot day #1	• 480 Switchboard training sentences<br>• 75 Moses evaluation sentences
9	2022.05.26 (57)	Real-time decoding pilot day #2	• 480 Switchboard training sentences<br>• 50 Moses evaluation sentences
10	2022.06.02 (64)	Real-time decoding pilot day #3	• 520 Switchboard training sentences<br>• 25 Switchboard evaluation sentences<br>• 50 Moses evaluation sentences
11	2022.06.07 (69)	Real-time decoding pilot day #4	• 480 Switchboard training sentences<br>• 40 Switchboard evaluation sentences<br>• 50 Moses evaluation sentences

12	2022.06.14 (76)	Real-time decoding pilot day #5	• 440 Switchboard training sentences<br>• 40 Switchboard evaluation sentences<br>• 50 Moses evaluation sentences
13	2022.06.16 (78)	Real-time decoding pilot day #6	• 440 Switchboard training sentences<br>• 40 Switchboard evaluation sentences<br>• 50 Moses evaluation sentences
14	2022.06.21 (83)	Real-time decoding pilot day #7	• 400 Switchboard training sentences<br>• 40 Switchboard evaluation sentences<br>• 50 Moses evaluation sentences
15	2022.06.23 (85)	Real-time decoding pilot day #8 (silent speaking)	• 440 Switchboard training sentences<br>• 80 Switchboard evaluation sentences<br>• 50 Moses evaluation sentences
16	2022.06.28 (90)	Real-time decoding pilot day #9	• 440 Switchboard training sentences<br>• 80 Switchboard evaluation sentences<br>• 50 Moses evaluation sentences
17	2022.07.05 (97)	Real-time decoding pilot day #10	• 400 Switchboard training sentences<br>• 80 Switchboard evaluation sentences<br>• 50 Moses evaluation sentences
18	2022.07.14 (106)	Real-time decoding pilot day #11	• 440 Switchboard training sentences<br>• 80 Switchboard evaluation sentences<br>• 50 Moses evaluation sentences
19	2022.07.21 (113)	Real-time decoding evaluation day (vocal #1)	• 440 Switchboard training sentences<br>• 80 Switchboard evaluation sentences<br>• 50 Moses evaluation sentences

20	2022.07.27 (119)	Real-time decoding evaluation day (vocal #2)	• 440 Switchboard training sentences<br>• 80 Switchboard evaluation sentences<br>• 50 Moses evaluation sentences
21	2022.07.29 (121)	Real-time decoding evaluation day cut short due to equipment failure (training data only)	• 240 Switchboard training sentences
22	2022.08.02 (125)	Real-time decoding evaluation day (vocal #3)	• 440 Switchboard training sentences<br>• 80 Switchboard evaluation sentences<br>• 50 Moses evaluation sentences
23	2022.08.11 (134)	Real-time decoding evaluation day (vocal #4)	• 260 Switchboard training sentences<br>• 80 Switchboard evaluation sentences<br>• 50 Moses evaluation sentences
24	2022.08.13 (136)	Real-time decoding evaluation day (vocal #5)	• 260 Switchboard training sentences<br>• 80 Switchboard evaluation sentences<br>• 50 Moses evaluation sentences
25	2022.08.18 (141)	Real-time decoding evaluation day (silent #1)	• 400 Switchboard training sentences<br>• 80 Switchboard evaluation sentences<br>• 50 Moses evaluation sentences
26	2022.08.23 (146)	Real-time decoding evaluation day (silent #2)	• 480 Switchboard training sentences<br>• 80 Switchboard evaluation sentences<br>• 50 Moses evaluation sentences
27	2022.08.25 (148)	Real-time decoding evaluation day (silent #3)	• 480 Switchboard training sentences<br>• 80 Switchboard evaluation sentences<br>• 50 Moses evaluation sentences

1.7. Instructed delay tasks

All tasks employed an instructed delay paradigm, with each trial consisting of an instructed delay phase followed by a go phase. For sentence speaking blocks, during the delay period the text of the sentence was displayed on the screen above a red square, providing T12 time to read it and prepare to speak. After the delay period, the red square cue then turned green, and the sentence remained on the screen while T12 attempted to speak it (either aloud or by silently mouthing it, depending on the session). When T12 finished speaking the sentence, she pushed a button held in her lap, which triggered the system to move to the next sentence trial.

For the single phoneme task, each phoneme was cued during the delay period with both text and an audio sample of that phoneme being spoken. Vowels were spoken in isolation and cued with the text of a word containing that vowel, with the vowel capitalized (e.g., strUt for ʌ). Consonants were all paired with the vowel "ah" following the consonant (denoted "AA" in ARPAbet notation and "ɑ" in IPA). Consonants were paired with a text cue evoking the sound of that consonant (e.g. "kah" or "wah"). For the single word task, where T12 spoke individual words from the 50-word Moses et al. word set [46], each word was cued with text only. For the orofacial movement sweep task, each movement was cued with a text description of that movement (e.g., "Tongue Up").

We ran a single "diagnostic" block at the beginning of each speech decoding session. Data from the diagnostic block was used to set electrode thresholds and linear regression reference (LRR) coefficients, and was also used to examine the rate of change of neural tuning across days. In this block, T12 spoke individual words from a diagnostic set of 7 words designed to span the space of articulation (with 8 repetitions per word). Words were cued with text only. The word set consisted of the following words: 'bah', 'choice', 'day', 'kite', 'though', 'veto', 'were'.

Task	Delay Low	Delay Mean	Delay High	Go Period	Return Period
Sentences	4.0 s	4.5 s	5.0 s	Variable (button-controlled)	None
Phonemes	1.8 s	2.3	2.8	1.7 s	None
Orofacial movements	2.0 s	2.5	3.0	1.0 s	1.0 s
Single words (50-word set)	2.0 s	2.5	3.0	2.0 s	None
Diagnostic block (7 words)	1.5 s	2.0	2.5	2.0 s	None

Table 3. *Task timing parameters.*

Delay period durations were pseudorandomly drawn from an exponential distribution with a task-specific mean (see Table 3); values that fell outside of a specified task-specific range were re-drawn. Go period durations were set fixed to a task-specific value for non-sentences tasks (for the sentence production task, T12 advanced the trial by pressing a button). Finally, in the orofacial movement sweep task, the go period was followed by a short "return" phase where T12 relaxed back to a neutral posture before starting the next trial. In all other tasks, the next trial started immediately after the go period ended.

1.8. Voiced vs. silent speaking behavior

For most speaking sessions, T12 was instructed to attempt to produce voiced speech in a "typical" manner (i.e., by trying to move all of her articulators and modulate her larynx to pass sound as one would to attempt to speak normally). The acoustic output was largely unintelligible. During the course

of the study, T12 reported that due to her reduced breath control abilities, attempting to produce voiced speech was fatiguing. We experimented with different speaking behavior paradigms and found that "mouthing" or silent speaking yielded similar decoding performance to voiced speech while being less fatiguing for T12. For silent speech sessions, we instructed T12 to pretend that she was mouthing the sentence to someone across the room. During this silent speaking behavior, T12 produced no audible sound, but visibly moved her lips, tongue and jaw. Sessions 15, 25, 26 and 27 were all performed with this silent speaking behavior.

1.9. Decoder evaluation sessions

Real-time speech decoding was evaluated in sessions 19, 20, 22, 23 and 24 for voiced speaking behavior and sessions 25, 26 and 27 for silent speaking behavior. These were the evaluation sessions reported in Fig. 2 and were conducted with the final version of the real-time decoder and parameters. Previous real-time decoding sessions were pilot sessions used to explore different online approaches and parameters.

Each evaluation session began with a "diagnostic" block as described previously (section 1.7). This block was used to calculate the threshold values and filters for online LRR that would be used for the rest of the session. Then, we collected "open-loop" blocks of sentences (~6 blocks with 40 sentences per block) during which no decoder was active. We trained the decoder using these blocks of data (combined with data from all past sessions) to obtain a "stage 1" RNN decoder. Next, we collected additional training blocks with real-time feedback, where the decoder output from the stage 1 RNN was displayed in real-time as T12 attempted to speak each sentence. Upon completion of each sentence, T12 would push a button to indicate she was finished and the decoded text was read aloud using Google Cloud's Text-To-Speech functionality. After the response was voiced by the computer, T12 pushed the button again to continue to the next sentence. Stage 1 real-time decoding was done for 3-6 blocks, with 40 sentences per block. Finally, the RNN was retrained a second-time using all open-loop and stage 1 data (combined with data from all past sessions) to yield a "stage 2" RNN. The stage 2 RNN was then evaluated on the 50 sentences from Moses et al 2021 [46] as well as 80 randomly-selected sentences from the Switchboard corpus, for which the final error rate and word per minute values were reported.

1.10. Sentence selection

For the first four initial training data collection sessions, sentences were randomly drawn from the OpenWebText2 corpus [47]. For all subsequent sessions, training sentences were drawn from the Switchboard corpus of telephone conversations between speakers of American English [48]. Sentences were selected by first generating lists of potential sentence segments by automatically splitting the transcription using provided punctuation marks. Sentence segments were then filtered to include only those that expressed a complete meaning, and superfluous starter words (e.g. "and") were deleted. We also did not include sentences with confusing or distracting meaning, such as violent or offensive topics. Finally, we upsampled sentences with rare phonemes to ensure there was sufficient training data for the RNN to learn these rare phonemes. This resulted in a diverse sample of sentences from spoken conversational contexts.

For each evaluation day, after the final "stage 2" RNN was trained, three evaluation blocks were run. These included one block of the 50 sentences used for evaluation in Moses et al 2021 [46] (these sentences were the same every session), and two blocks of 40 sentences each from Switchboard, selected in the same manner as the training data. The Switchboard sentences were different for each evaluation session. The RNN decoder was never evaluated on a sentence that it had been trained on, and every sentence was unique (except for the "direct comparison" blocks that always used the same 50 sentences from Moses et al 2021). When we retrained the decoder each day before performance evaluation, we retrained it using all previously collected data (from all prior days) except for these direct comparison blocks, in order to prevent the RNN from overfitting to these repeated sentences.

2. Neural representation of orofacial movements and speech in orofacial cortex

2.1. Tuning heat maps

To generate the neural tuning heat maps shown in Figure 1, we first started with binned threshold crossing spike counts using a -4.5 RMS threshold (20 ms bins). To account for drifts in mean firing rates across the session, the binned threshold crossing rates were mean-subtracted within each block (i.e., for each electrode, its mean firing rate within each block was subtracted from each time step's binned spike count).

Next, for each trial and electrode, threshold crossing counts were averaged in an 800 ms window (200 to 1000 ms after the go cue). Significance of tuning was then assessed via 1-way ANOVAs applied per electrode, where each ANOVA group corresponded to a different movement condition of a given movement type, and each observation was a scalar average firing rate for a single trial. The movement conditions that were used to assess tuning to each movement type are shown in Extended Data Fig. 3. P-values from each ANOVA were used to define tuning significance (p<1e-5) for the tuning heatmaps (Fig 1f) and mixed tuning counts (Extended Data Fig. 4). To make the statistical power of the "phonemes" and "words" movement types comparable to the orofacial movement types (so that the amount of tuning to phonemes/words can be fairly compared to orofacial movement), we used only the following 6 phoneme or word conditions as opposed to using all 39 phonemes and all 50 words: (B, HH, N, TH, Z, IY) and (am, coming, good, hungry, no, tell). The results appeared robust to the particular set of phonemes/words chosen.

The fraction of variance accounted for by movement tuning on a single electrode was defined as:

$$FVAF = 1 - \frac{SSERR}{SSTOT}$$

SSTOT is the total sum of squared average firing rates over all trials. For computing SSTOT, squaring was performed after the grand mean across all trials was subtracted from each trial first, so that the overall mean firing rate did not contribute to the variance.

SSERR is the sum of squared prediction errors across all trials. Prediction error was assessed with a cross-validated (5-fold) model which predicts the firing rate of each trial based only on the mean of the condition it belongs to. Condition-specific means were estimated on the training set by taking the sample means across training trials, and then applied to the held-out test set.

If there are large differences in mean firing rate between movement conditions (i.e., strong movement tuning), then SSERR will be small relative to SSTOT. Cross-validation prevents overestimation of tuning due to spurious differences in mean firing rate between conditions that are not stable across folds.

2.2. Naive Bayes classification

Offline classification results (reported in Fig 1d,e and Extended Data Fig. 3) were generated using a cross-validated (leave-one-out) Gaussian naive Bayes classifier, following the methods described in [49], using threshold crossing rates computed in a window from 0 to 1000 ms after the go cue (Fig 1d, Extended Data Fig. 3) or a sliding 100 ms window (Fig 1e). We used -4.5 x RMS thresholds for classification. 95% confidence intervals for classification accuracies were computed with bootstrap resampling (10,000 resamples). We chose to use a Gaussian naive Bayes classifier because it is a simple method that performed well enough to demonstrate the existence of strong neural tuning - it is likely that more advanced methods could improve classification accuracy further.

2.3. Preserved articulatory representation of phonemes

2.3.1. Electromagnetic articulography (EMA) representations

Electromagentic articulography (EMA) and corresponding audio data was taken from the publicly available "USC-Timit" dataset [50] and the "Haskins Production Rate Comparison" dataset [51], which both contain phoneme labels (the beginning and end of each phoneme is marked for each sentence). EMA data was collected with markers placed on the lip, tongue and jaw. We used only the X and Y positions of each marker (sagittal plane position), yielding an articulatory time series with dimensionality ranging from 12 (6 markers) to 16 (8 markers) depending on the subject.

To compute an EMA representation of each phoneme, we averaged over all EMA marker position data recorded at time points that were labeled as belonging to that phoneme, yielding a single average articulator position vector for each phoneme. Finally, a binary voicing indicator variable was added to the EMA representation of each phoneme. This variable was set to 1 for voiced phonemes (e.g., 'z') and 0 for unvoiced phonemes (e.g., 's'). Since EMA does not measure voicing, this is a simple way to include some voicing information that would otherwise be omitted from the EMA representation.

EMA data from USC-Timit subject 'M1' was used for all Figure 3 analyses. Extended Data Fig. 8 shows data from all 12 subjects.

2.3.2. Saliency vector representation

Saliency vectors, which quantify the neural vectors which maximally excite each of the decoder's phoneme outputs, were generated by computing RNN logit gradients with respect to the input features. We used an RNN trained on all voiced speech days and then used the first 30 trials from each day's test set to compute the gradients.

For each day's data, we run the RNN over each sample

$$x^{(t)} \in \mathbb{R}^{256}$$

for five time steps (first initializing the hidden state to zeros) - this allows the network some time to integrate information about the specific feature vector. This yields a phoneme probability output for each time step

$$f_{RNN}(x^{(t)}) = y^{(t)} \in \mathbb{R}^{41}$$

For each time step t, we then calculate the Jacobian matrix J, which contains entries corresponding to first-order partial derivatives of each logit output with respect to each channel, i.e.

$$J_{i,:} = \nabla y_i^{(t)}|_{x^{(t)}}$$

This gradient records how small changes in each channel's activity influences the probability of class i. We can calculate the Jacobian for all phonemes and timesteps, resulting in a time x channels x phonemes matrix M where

$$M_{t,:,:}$$

contains the Jacobian at timestep t. We then average across the time dimension to obtain an integrated estimate of how each channel's activity influences different phoneme class probabilities.

To compute the gradients, we used SmoothGrad [52], a method for denoising saliency maps by computing gradients over an input with multiple noise perturbations, i.e.

$$\nabla y_i^{(t)}|_{x^{(t)}} + N(0, \sigma)$$

The resulting saliency map estimates are then averaged together. We use n = 20 perturbations and noise level = 10 (relative to the overall range of firing rates after capping outliers). This extra step contributed a small but consistent improvement in similarity matrix correlations with the EMA data.

2.3.3. Similarity matrices

Similarity matrices in Figure 3a and 3d were computed using cosine similarity. That is, for each pair (x, y) of RNN saliency or EMA vectors, similarity was defined as

$$\frac{x \cdot y}{||x||||y||}$$

This equation computes the cosine of the angle between x and y. Before computing cosine similarity, the vectors were first centered by subtracting the mean across all consonant (Figure 3a) or all vowel vectors (Figure 3d).

2.3.4. Correlation between articulatory and neural representations

Figure 3b and 3e show the correlation between the neural and EMA phoneme representations, which was computed across all phonemes and dimensions after a cross-validated Procrustes alignment of the saliency vectors to the EMA vectors. In Extended Data Fig. 8, this same method was used to compute the correlation between neural representations and articulatory representations that were estimated in different ways. In Extended Data Fig. 8c, correlation values were computed between consonants and vowels separately and then averaged together to produce a single value.

First, neural vectors were concatenated into a 256 x 39 matrix **X** (256 neural features x 39 phonemes) and articulatory vectors were concatenated into a N x 39 matrix **Y** (N articulatory dimensions x 39 phonemes). **X** and **Y** were then reduced to D dimensions using PCA applied across the rows, yielding an D x 39 matrix $\tilde{\mathbf{X}}$ and an D x 39 matrix $\tilde{\mathbf{Y}}$. D was set to 12 if N>=12, otherwise D was set equal to N.

$\tilde{\mathbf{X}}$ was then aligned to $\tilde{\mathbf{Y}}$ using a cross-validated orthogonal rotation (Procrustes analysis) using leave-one-out cross-validation. Specifically, for each vector x_i in $\tilde{\mathbf{X}}$, Procrustes analysis was applied to align all other vectors $\{x_1, ...x_{i-1}, x_{i+1}, ...x_n\}$ to the matching vectors $\{y_1, ...y_{i-1}, y_{i+1}, ...y_n\}$, yielding an orthogonal rotation **R**. **R** was then applied to x_i to yield $\hat{x}_i$. Orthogonality enforces that the rotation be rigid, so that the underlying structure in the data is preserved.

Finally, all $\hat{x}_i$ vectors were concatenated into an (8x39) x 1 vector $\bar{x}$ and all $\tilde{y}_i$ vectors were concatenated into an (8x39) x 1 vector $\bar{y}$. The Pearson correlation coefficient was then calculated between $\bar{x}$ and $\bar{y}$ using consonant entries only (Figure 3b) or vowel entries (Figure 3e).

As a control, this same procedure was repeated 10,000 times but with the columns of **X** shuffled into a random order, which allows estimation of what the correlation could be expected to be 'by chance' if each phoneme's vector was random but drawn from the same distribution. Note that the cross-validation procedure causes the chance distribution to be centered at 0 (otherwise it would be biased upwards as Procrustes would overfit and align noise). The true correlation is far greater than any of the 10,000 shuffle results, indicating statistical significance.

2.3.5. Low-dimensional visualization of phoneme geometry

To make the plots in Figure 3c and 3f, the neural saliency vectors were first aligned to the EMA vectors using cross-validated Procustes analysis, as described in the above section. After alignment, the top two dimensions were plotted; for vowels, these two dimensions were rotated and flipped within the plane in order to highlight the classic (front vs. back) and (high vs. low) structure.

2.3.6. Place and formant representation

In Extended Data Fig. 8, we tested a different method for quantifying the articulatory representation of phonemes based on classical ways of understanding consonants and vowels. We represented each consonant based on its place of articulation (labial, dental, alveolar, post-alveolar, velar, glottal) by using a 24 x 6 "place code" matrix. In this matrix, each row is a consonant and each column corresponds to one of the six places of articulation. For each consonant, we placed a "1" in the column corresponding to its place of articulation and a "0" in all other columns. We represented vowels using a 15 x 2 "formant"

matrix. In this matrix, each row is a vowel, and the two columns correspond to the logarithm of the first and second formant frequencies [53].

2.3.7. Decoder labeling representation

In Extended Data Fig. 8, we tested different methods for quantifying the neural representation of phonemes. One of these was the "Decoder Labels" method, which was based on identifying time steps that belonged to each phoneme using the RNN decoder output, and then averaging across these time steps to find a neural representation vector for each phoneme.

To accomplish this, we used the segmentation algorithm from [54]. This algorithm estimates a time window during each phoneme occurs, given the outputs of a decoder trained with the CTC loss function [55]. For this approach, we trained a bidirectional RNN offline, because previous studies have found that bidirectional RNN strained with the CTC loss produces better alignment than unidirectional RNNs [56, 57]. Because RNN models trained with the CTC loss tend to delay their predictions [56, 57], we extended the start time of each phoneme's neural activity segment by one kernel size (defined in Table 5). Once the time windows for each phoneme were identified, we simply averaged over all time steps to generate a single average neural vector for each phoneme. For offline RNN training, we followed the procedure described below in section 7.

2.3.8. Single phoneme task representation

In Extended Data Fig. 8, we tested different methods for quantifying the neural representation of phonemes. One of these was the "Single Phoneme Task" method, which was based on the instructed delay task shown in Fig. 1. In this task, T12 spoke individual vowels or consonants paired with the same vowel "AA". To compute the neural representation of each phoneme, we simply averaged the threshold crossing rates within a 0 to 1000 ms window after the go cue for each trial belonging to that phoneme (threshold = -4.5 RMS).

2.4. Neural correlation across days

To compute how the neural representation of speech was correlated between pairs of days (Fig. 4d), we used data from a "diagnostic block" collected at the beginning of each day. During this block, T12 completed an instructed delay task where she attempted to speak individual words from a set of 7 words designed to span the space of articulation (8 repetitions per word). The word set consisted of the following words: 'bah', 'choice','day','kite','though','veto', and 'were'. We also included a condition where T12 was instructed to rest silently ('do nothing').

First, threshold crossing rates for each trial were averaged between a 100 to 600 ms window after the go cue to yield a single firing rate vector for each trial (of length 128). Then, "pseudo-trial" vectors were created by concatenating together a single firing rate vector from each condition, resulting in pseudo-trial vectors of length 128*8=1024. The result of this step is a set of eight vectors $\{v_1, v_2, ..., v_8\}$, one vector for each of the eight repetitions of all conditions. When assessing the similarity between any two days, we then have two sets of vectors to consider: $\{v_1, v_2, \ldots, v_8\}$ and $\{u_1, u_2, \ldots, u_8\}$. Consider each of these vectors as a random draw from a day-specific distribution (let us denote the two distributions as V and U). To quantify similarity, we estimated the correlation between the means of V and U (note that the means themselves are also vectors). The quantity of interest here is the mean because this represents the average firing rates observed for each condition (i.e., the neural representation of each word). To estimate the correlation between the means of V and U, we used a cross-validated measure of correlation that reduces the impact of noise. See our prior work [49] and accompanying code repository https://github.com/fwillett/cvVectorStats for more details about this method. Importantly, this cross-validated method is different from simply correlating $\frac{1}{8}\sum_{i=1}^{8} v_i$ and $\frac{1}{8}\sum_{i=1}^{8} u_i$, which would underestimate the true correlation due to noise that causes the estimated means to appear more dissimilar

than they really are. For example, even if V and U have identical means, noise in v_i and u_i would always cause the estimated correlation to be less than 1 when correlating $\frac{1}{8}\sum_{i=1}^{8} v_i$ and $\frac{1}{8}\sum_{i=1}^{8} u_i$ together.

To make the plot in Fig. 4d, we included all pairings of the following 12 days on which a diagnostic block of attempted vocal speaking was collected: 2022.06.16, 2022.06.21, 2022.06.28, 2022.07.05, 2022.07.07, 2022.07.14, 2022.07.21, 2022.07.27, 2022.07.29, 2022.08.02, 2022.08.11, 2022.08.13.

3. Phoneme transcription and labelling

Each sentence prompt was transcribed into a sequence of phonemes using the CMU Pronouncing Dictionary (http://www.speech.cs.cmu.edu/cgi-bin/cmudict) and the g2p software package [58]. The CMU Dictionary uses 39 phonemes, each denoted using the ARPAbet symbol set developed for speech recognition (see Table 4 for the correspondence between IPA notation and ARPAbet notation, and https://en.wikipedia.org/wiki/ARPABET). Note that we did not incorporate the stress labeling given by the CMU dictionary for vowels (i.e., we labeled each vowel in the same way regardless of how it is stressed in the word).

We automatically added a "silence" phoneme to the end of each word in order to denote the separation between words. We reasoned that this gives the RNN decoder the ability to communicate demarcations between words to the language model, and we found that it improved performance. Note that the silence token is not necessarily intended to model literal silence, as the participant may or may not be silent between each word (although she appeared to take brief pauses between many of the words, as can be seen in SVideo 1).

4. Decoder performance metrics

4.1. Word and phoneme error rates

We evaluate both phoneme error rate and word error rate. Phoneme error rate was defined as the edit distance between the decoded sequence of phonemes and the prompt sentence phoneme transcription (i.e., the number of insertions, deletions or substitutions required to make the sequence of phonemes match exactly). Similarly, word error rate was the edit distance defined over sequences of words.

Note that the reported error rates are "aggregate" error rates, which are the result of combining across many independent sentences. To combine data across multiple sentences, we summed the number of errors across all sentences and divided this by the total number of phonemes/words across all sentences (as opposed to computing error rates first for each sentence separately, and then averaging the rates). This helps prevent very short sentences from overly influencing the result.

Confidence intervals for error rates were computed via bootstrap resampling over individual trials and then re-computing the aggregate error rates over the resampled distribution (10,000 resamples).

4.2. Words per minute

Words per minute was defined as the number of words spoken divided by the total amount of speaking time. Speaking time for each trial was defined as the time from which the cue turned green to when the participant pushed the button to signal she had completed saying the prompted sentence. The speaking rates reported in Figure 2c are "aggregate" rates which were computed by first summing the total number of words spoken across all trials, and then dividing by the total speaking time across all trials (as opposed to computing a speaking rate for each sentence separately and then averaging the rates). This helps prevent very short sentences from overly influencing the result.

Confidence intervals for words per minute were computed via bootstrap resampling over individual trials and then re-computing the aggregate speaking rate over the resampled distribution (10,000 resamples).

ARPAbet Notation	IPA Notation	Example
AA	ɑ	bot
AE	æ	bat
AH	ʌ	but
AO	ɔ	caught
AW	aʊ	bout
AY	aɪ	bite
EH	ɛ	bet
ER	ɝ	bird
EY	eɪ	bait
IH	ɪ	bit
IY	i	beat
OW	oʊ	boat
OY	ɔɪ	boy
UH	ʊ	book
UW	u	boot
B	b	buy
CH	ʧ	China
D	d	die
DH	ð	thy
F	f	fight
G	ɡ	guy
HH	h	high
JH	ʤ	jive
K	k	kite
L	l	lie
M	m	my
N	n	nigh
NG	ŋ	sing
P	p	pie
R	ɹ	rye
S	s	sigh
SH	ʃ	ship
T	t	tie
TH	θ	thigh
V	v	vie
W	w	wise
Y	j	yacht
Z	z	zoo
ZH	ʒ	pleasure

Table 4. *ARPAbet and IPA correspondence.*

5. RNN architecture

We used a 5 layer, stacked gated recurrent unit RNN [59] to convert T12's neural activity into a time series of phoneme probabilities. The RNN ran at a 4-bin frequency (20 ms bins), outputting a phoneme probability vector every 80 ms. A 14-bin window of neural activity was stacked together and fed as input to the RNN at each 80 ms cycle (in other words: kernel size = 14, stride = 4). See Extended Data Fig. 5 for parameter sweeps that justify these and other architecture choices.

5.1. Feature pre-processing and day-specific input layers

Threshold crossing rates and spike band power features were pre-processed by binning into 20 ms time steps, "z-scoring" (mean-subtracted and divided by the standard deviation), causally smoothed by convolving with a Gaussian kernel (sd = 40ms) that was delayed by 160ms, concatenated into 256 x 1 vector, and then transformed using a day-specific input layer. Z-scoring was performed using block-specific means and standard deviations (to account for non-stationarities in the features that accrue over time across blocks). Using day-specific input layers outperformed the alternative of a shared input layer across all days (Extended Data Fig. 5B).

The day-specific input layers consisted of an affine transformation applied to the feature vector followed by a softsign activation function:

$$\tilde{x_t} = \text{softsign}(Wx_t + b) \tag{1}$$

Here, $\tilde{x_t}$ is the day-transformed input vector at time step t, W_i is a 256 x 256 matrix and b_i is a 256 x 1 bias vector for day i, and the softsign function is applied element-wise to the resultant vector (where $\text{softsign}(x) = \frac{x}{|x|+1}$). W_i and b_i were optimized simultaneously along with all other RNN parameters. During training, dropout was applied both prior to and after the softsign.

5.2. Rolling z-scoring

During online evaluation, we used a rolling estimate of the mean and standard deviation of each feature to perform z-scoring. This helps account for neural non-stationarities that accrue across time, and substantially outperforms the alternative of using the prior block's means and standard deviations (Extended Data Fig. 5A).

For the first ten sentences of a new block, we used a weighted average of the prior block's mean estimate and the mean of whatever sentences were collected so far in the current block:

$$u_i = \frac{11-i}{10} * u_{prev} + \frac{i-1}{10} * u_{curr} \tag{2}$$

Here, u_i is the mean used to z-score sentence i, u_{prev} is the prior block's mean estimate, and u_{curr} is the mean across all sentences collected so far in the current block. After ten sentences had been collected, we stopped incorporating the prior block's mean and simply took the mean across the most recent $\min(20, N)$ sentences, where N is the number of sentences collected so far in the current block. The standard deviation was updated in the same way as the mean.

6. RNN training overview

6.1. Connectionist temporal classification (CTC) loss

Due to T12's inability to produce intelligible speech, we had no ground truth labels of what phonemes were being spoken at each time step. The lack of ground truth labels makes it difficult to apply simple supervised training techniques to train the RNN. To get around this problem, we used the Connectionist Temporal Classification (CTC) loss function, which can train neural networks to output a sequence of symbols (in this case, phonemes) given unlabeled time series input [55]. Using the CTC loss function results in an RNN that is trained to output a time series of phoneme probabilities (with an extra "blank" token probability). A language model can then be used to infer a sequence of underlying words from these probabilities, or phonemes can be decoded from these probabilities simply by emitting the phoneme of maximum probability at each time step (while taking care to omit repeats and time steps where "blank" is the maximum probability).

6.2. Artificial noise

We added two types of artificial noise to the neural features to regularize the RNN. First, we added white noise directly to the input feature vectors at each time step. Adding white noise to the inputs asks the RNN to map clouds of similar inputs to the same output, improving generalization. We also added artificial constant offsets to the means of the neural features, to make the RNN more robust to non-stationarities in the neural data. Drifts in the baseline firing rates that accrue over time has been an important problem for intracortical BCIs [60, 61, 62]. The constant offset values were randomly chosen on each minibatch and were constant across all time steps in the minibatch, but unique to each feature.

The two above-mentioned types of noise (white noise and constant offset noise) were combined together to transform the input vector in the following way:

$$x_t' = x_t + \epsilon_t + \phi \quad (3)$$

Here, x_t' are the neural features with noise added, x_t are the original neural features, ϵ_t is a white noise vector unique to each time step, and ϕ is a constant offset vector.

6.3. Supervised training

The RNN was implemented with TensorFlow 2 and trained using stochastic gradient descent (ADAM; $\beta_1 = 0.9$, $\beta_2 = 0.999$, $\epsilon = 0.1$) for 10,000 minibatches (batch size = 64). The learning rate was decayed linearly from 0.02 to 0.0 across the 10,000 minibatches. We applied dropout and L2 weight regularization during training to improve generalization. See Table 5 for a list of RNN hyperparameters.

RNN Parameter	Description	Value
nUnits	Number of units in each GRU layer	512
nLayers	Number of GRU layers	5
Kernel Size	Number of input feature time bins stacked together as a single input for the RNN	14
Stride	Describes how many time bins the RNN skips forward every step	4
L2	L2 regularization cost	1e-0.5
Dropout	Probability of dropout during training	0.4
WhiteNoiseSD	Standard deviation of white noise added to input data for regularization	1.0
constantOffsetSD	Standard deviation of constant offset noise added to input data to improve robustness against non-stationary feature means	0.2
Batch Size	Number of sentences included in each mini-batch	64
Learning Rate	Linearly decaying learning rate	0.02 to 0.0
β_1	ADAM stochastic gradient descent parameter	0.9
β_2	ADAM stochastic gradient descent parameter	0.999
ϵ	ADAM stochastic gradient descent parameter	0.1

Table 5. *Architecture, training and regularization parameters for RNN Model.*

7. Offline performance sweeps

7.1. Overview

To determine the effect of different design choices made for the RNN architecture, and to understand the impact of data quantity and channel count, we performed several performance sweeps offline (results from this are shown in Extended Data Fig. 5, Extended Data Fig. 7 and Fig 4). Unless otherwise specified, we trained 10 seeds of an RNN model for each variation of parameters and performed inference on a standardized set of held-out test data. To define the training/held-out set (called the "Proximal Test Set" in Table 1 from the main text), we took 50 sentences at random from each day as the held-out set and used the remaining sentences as offline training data, drawing from all open-loop/stage 1 sentences on each day (but excluding stage 2 evaluation data). We used the original language model that was run online (as opposed to the improved version reported in Table 1).

We ran parameter sweeps for the GRU-RNN architecture choices including number of units, number of layers, kernel size and stride (Extended Data Fig. 5e-h). A comparison between a shared input network and unique input network per session was also run (Extended Data Fig. 5b). Furthermore, performance when using different kinds of features was also compared in this manner (Extended Data Fig. 5c-d), including four different threshold crossing thresholds (-3.5,-4.5,-5.5,-6.5), spike band power, area 44 vs. area 6v features, and the mel-frequency cepstral coefficients (MFCCs) of the participants' recorded audio during attempted speaking sessions. MFCCs were computed using 40 ms window (using MATLAB 2020a's "mfcc" function).

7.2. Effect of channel count on performance

To determine the effect of channel count on decoding performance (Fig 4b), 100 seeds of each multiple of 10 number of channels up to the full 128 channels was run. For each of the 100 seeds for each channel count, channels were randomly selected without replacement. To predict performance for higher number of channels past 128, a least squares linear regression was fit to the log-log relationship of the number of channels vs. error rate.

7.3. Amount of training data

To plot the number of days of training data versus performance (Extended Data Fig. 5i), RNNs were trained for each of the 5 vocal speaking evaluation days separately, and for each number of training data days going consecutively in reverse until all previous days were used in training. Performance was assessed only on the given evaluation day. Word error rates were then averaged over all evaluation days to produce a single (# of days) vs. (word error rate) curve.

We also tested whether or not it was necessary to retrain the RNN decoder on each new performance evaluation day using hundreds of new sentences collected on that day, or whether fewer (or no) new sentences might have also yielded good performance, which would be a more realistic use case (Fig 4c). For this analysis, models were trained on the five attempted speech evaluation sessions (sessions 18,19,21,22,23) using reduced subsets of sentences from the given evaluation day (while still using all historical data). The input layer for each given evaluation day was also tied to be the same as the most recent historical day, in order to prevent overfitting when using a small number of training sentences. Once trained, RNNs were evaluated on the same set of "stage 2" online evaluation sentences used to report performance in Figure 2.

7.4. Language model vocabulary size sweep

To test how the number of words in language model (LM) affects the decoding accuracy, we built different 3-gram LMs with various vocabulary sizes. These LMs were built following the same procedure as in Section 8, but with vocabulary sizes varying from 50 to 140,000. We started with the 50 words from

[46], and gradually added words until the vocabulary size reached 140,000. The added words were chosen from the LM training corpus. Words were added in the order of their frequencies in the training corpus. When the vocabulary size became greater then 4500, we pruned the LM with threshold $1e-9$. To measure the WER, we ran the LM decoders on the CTC probabilities output by RNN from the 8 real-time speech decoding sessions (19, 20, 22, 23, 24, 25, 26, and 27).

8. Language model

8.1. Overview

We used a n-gram language model (LM) to decode word sequences from RNN outputs for real-time decoding and offline analyses. Here, we give an overview of the major steps involved. The n-gram LM was created with Kaldi [63] using OpenWebText2 corpus [47]. We first preprocessed the text corpus to only include English letters and limited punctuation marks. Then we used Kaldi to construct a n-gram LM, using either the CMU Pronunciation Dictionary [1] (125k words) or the 50 words from [46]. The LM was represented in the form of a weighted finite-state transducer [64] which can be used to translate the a sequence of CTC labels into candidate sentences.

8.2. OpenWebText2 preprocessing

Our n-gram LM was created using samples from OpenWebText2 [47]. OpenWebText2 is a text corpus covering all Reddit submissions from 2005 up until April 2020. We downloaded the entire corpus and randomly sampled 95% as a training corpus. We preprocessed the training corpus to include only English letters and 4 punctuation marks (period, comma, apostrophe, and question mark). The preprocessed corpus was then split into sentences and converted to upper case (yielding a total of 634M sentences with 99B words).

8.3. Constructing the n-gram language model

We used publicly available scripts[2] as a starting point for constructing our n-gram LM. The script first uses SRILM [65] to count the frequencies of n-grams (unigram, bi-gram, and 3-gram, etc.) in the training corpus. We used the Good-Turing discounting method [66] to improve probability estimation of unseen or rare word combinations. For words that are not in the pronunciation dictionary, they are mapped to a special token <UNK>. When using the CMU Pronunciation Dictionary, the resutling LM is too large to fit into the main memory of the Ubuntu computer used for real-time inference. We pruned the resulting n-gram LM using SRILM, which removes n-grams that causes the perplexity of the LM to increase by less than a threshold. The LM built with the 50 words from [46] is not pruned. For online real-time decoding, we used a 3-gram LM pruned with threshold $1e-9$. For offline analyses, we used a 5-gram LM pruned with threshold $4e-11$.

The n-gram LM was then converted to a weighted finite-state transducer (WFST) [64]. A WFST is a finite-state acceptor in which each transition has an input symbol, an output symbol and a weight. A path through the WFST takes a sequence of input symbols and emits a sequence of output symbols. We followed the recipe in [67] to construct our WFST search graph:

$$T \circ L \circ G \tag{4}$$

Here, $\circ$ denotes composition.

G is the grammar WFST that encodes legal sequences of words and their probabilities based on the n-gram LM.

[1] http://www.speech.cs.cmu.edu/cgi-bin/cmudict
[2] https://github.com/thu-spmi/CAT/blob/v1/egs/libri

L is the lexicon WFST that encodes what phonemes are contained in each legal word. Note that a "silence" phoneme was added to the end of each word (see section 3). We did an offline sweep of the "silence" phoneme probability and found 0.9 to be optimal.

Finally, T is the token WFST that maps a sequence of RNN output labels to a single phoneme. In our case, T contains all the individual phonemes plus the CTC blank symbol. For more details about how the three WFSTs were composed, refer to [67].

8.4. Inference with the n-gram language model

We used the LM decoder implementation in WeNet [68] for efficient real-time inference. WeNet is a wrapper around Kaldi to simplify the implementation of a real-time LM decoder. The LM decoder runs an approximate Viterbi search (beam search) algorithm on the WFST search graph to find the most likely sequences of words. The WFST search graph encodes the mapping from a sequence of CTC labels emitted by RNN to a sequence of words. During inference, the beam search combines information from the WFST (state transition probabilities) and information from the RNN decoder about which CTC labels are likely occurring at each moment in time. We do not normalize the CTC label probabilities as in [67].

The decoding parameters for beam search in defined in Table 6. The beam search runs every 80ms, after the RNN emits CTC label probabilities. On average, each beam search step took less than 1ms to complete.

8.5. Offline language model optimization

After data collection was completed, we further optimized the LM and found that online decoding WER could have improved by 6.4% with an improved LM architecture (Table 1 in main text). To improve the LM, we used a 5-gram LM instead of 3-gram LM and employed a 2-pass decoding strategy. The first pass of the 2-pass decoder is the same as the 1-pass decoder described above. But instead of outputting a decoded sentence, it outputs a word lattice [69, 70]. A word lattice is a directed graph where each node is a word and the an edge between nodes encodes the transition probability between words. It is a efficient representation to encode possible word sequences. The second pass of the 2-pass decoder uses a unpruned n-gram LM to rescore the word lattice. Rescoring replaces the original LM score with a more accurate score from the unpruned LM. After rescoring, we pick the best path through the word lattice as decoding output.

Finally, we found that using a transformer LM [71] to rescore the candidate sentences in an third pass could further improve decoding accuracy. Transformer LMs have been the state of the art in many natural language tasks in recent years [72, 73]. Compared to an n-gram LM which models a limited context (e.g., 3 words for a trigram model), a transformer LM can model much longer contexts (e.g., 1024 words). Training a transformer LM requires a significant amount of computation resources. We used the publicly available pre-trained OPT LM [74]. We used the largest OPT LM (6.7B parameters) that can fit into one NVIDIA A100 40GB GPU. The OPT LM was used to rescore the n-best outputs from a 2-pass decoder.

The 2-pass decoder first outputs at most n sentences with the highest decoding scores. The decoding score of a sentence was defined as follows:

$$score(s) = \alpha * log(P_{RNN}(s)) + log(P_{ngram}(s)) \tag{5}$$

Here $P_{RNN}(s)$ is the sentence s's corresponding CTC label sequence probability output by the RNN. P_{ngram} is the sentence s's probability estimated by the n-gram LM. α is the *acoustic scale* defined in Table 6.

We then used OPT to evaluate the probability of each sentence in the n-best list and linearly interpolate with the n-gram LM's probability. The new score function was defined as follows:

$$score(s) = \alpha * log(P_{RNN}(s)) + \beta * log(P_{ngram}(s)) + (1-\beta) * log(P_{opt}(s)) \quad (6)$$

Here $P_{opt}(s)$ is sentence s's probability estimation from OPT LM. β is the *lm weight* defined in Table 6. The top scored sentence is the final decoding output.

Finally, we found that decoding accuracy was improved by dividing the CTC blank label probability by a constant value [75], which adds a cost for not outputting any labels.

All LM decoding parameters are optimized via grid search on a validation data set (session 7-16).

**LM Decoding Parameter**	**Description**	**Value**
min active	Beam search decoder's minimum active states	200
max active	Beam search decoder's maximum active states	7000
beam	Beam size	17
acoustic scale	Scaling factor on RNN's log probabilities	0.8
lm weight	Interpolation weight between LMs	0.5
n-best	Number of decoding hypotheses	100
blank penalty	Penalty applied on blank labels	log(7)

Table 6. *LM decoding parameters .*

9. Statistics

Table 7 below lists statistical details for each confidence interval or hypothesis test reported in this work. In this study, uncertainty was quantified mainly with 95% confidence intervals computed using the bootstrap percentile method.

Result	Statistical Details
Figure 1	Mean firing rates in Figure 1c were taken over 20 trials (for orofacial movements and words) or 16 trials (for phonemes). 95% confidence intervals were estimated using bootstrap resampling over trials (10,000 resamplings). For Figure 1d, 95% confidence intervals were estimated by first computing a classification success or failure for each trial using leave-one-out cross-validation (660 orofacial movements trials, 1,000 word trials, and 720 phoneme trials). A confidence interval for classification accuracy was then computed by bootstrap resampling the success/failure observations for each trial (10,000). For Figure 1e, 95% confidence intervals were computed separately for each classification window in the same way as Figure 1d. For Figure 1f, one-way ANOVAs with a threshold of $p<1e\text{-}5$ were used to assess the presence of statistically significant tuning to each movement category on each electrode (see section 2.1). We had 20 trials for each movement, and each of the movement categories had the following number of movements: forehead (4), eyelids (4), jaw (6), lips (6), tongue (5), larynx (4), phonemes (6), words (6).
Figure 2	Circles in Figure 2b and 2c show the mean word error rates and words per minute for 80 evaluation trials (125k vocab) or 50 evaluation trials (50 word vocab) on a single day. Lines show the 95% confidence intervals estimated by bootstrap resampling (10,000 resamplings) over the 80 available trials (125k vocab) or 50 available trials (50-word vocab) trials.
Table 1	Online error rates in the table are computed across all evaluation trials (125k-word vocab: 400 trials for vocal speaking, and 240 trials for silent speaking; 50-word vocab: 250 trials for vocal, 150 trials for silent). Offline error rates for the proximal test set were computed across 250 held-out trials from the five evaluation days.
Figure 3	Statistical significance of the neural-articulatory correlations was assessed via a reshuffling control (10,000 reshufflings) - see section 2.3.4. The actual correlations we found were greater than those for all 10,000 reshufflings, indicating significance.
Figure 4	95% confidence intervals shown in Figure 4a were computed by bootstrap resampling across the 250 evaluation trials for the 50-word vocabulary. Standard deviations in Figure 4b were computed over 10 RNN seeds. 95% confidence intervals in Figure 4c were computed via bootstrap resampling over 10 RNN seeds.
Extended Data Fig. 4	Tuning to movement categories was assessed using a statistical significance threshold ($p<1e\text{-}5$) with a one-way ANOVA analysis, using the same methods we used to produce the tuning heatmaps in Figure 1f.
Extended Data Fig. 5	Standard deviations shown in Extended Data Fig. 5i were computed over 10 RNN seeds.
Extended Data Fig. 7	Word error rates in Extended Data Fig. 7a were aggregated across the 400 closed-loop evaluation trials for the 125k vocab collected for the 5 performance evaluation days.
Extended Data Fig. 8	Chance distributions shown in Extended Data Fig. 8b were computed using a shuffle control (10,000 reshufflings) - see section 2.3.4.

Table 7. *Statistical analyses.*

References

[39] Matthew F. Glasser, Timothy S. Coalson, Emma C. Robinson, Carl D. Hacker, John Harwell, Essa Yacoub, Kamil Ugurbil, Jesper Andersson, Christian F. Beckmann, Mark Jenkinson, Stephen M. Smith, and David C. Van Essen. A multi-modal parcellation of human cerebral cortex. *Nature*, 536(7615):171, August 2016. http://dx.doi.org/10.1038/nature18933. URL https://www.ncbi.nlm.nih.gov/pmc/articles/PMC4990127/.

[40] Nicolas Y. Masse, Beata Jarosiewicz, John D. Simeral, Daniel Bacher, Sergey D. Stavisky, Sydney S. Cash, Erin M. Oakley, Etsub Berhanu, Emad Eskandar, Gerhard Friehs, Leigh R. Hochberg, and John P. Donoghue. Non-causal spike filtering improves decoding of movement intention for intracortical BCIs. *Journal of Neuroscience Methods*, 236:58–67, October 2014. ISSN 0165-0270. http://dx.doi.org/10.1016/j.jneumeth.2014.08.004. URL https://www.sciencedirect.com/science/article/pii/S016502701400288X.

[41] D. Young, F. Willett, W. D. Memberg, B. Murphy, B. Walter, J. Sweet, J. Miller, L. R. Hochberg, R. F. Kirsch, and A. B. Ajiboye. Signal processing methods for reducing artifacts in microelectrode brain recordings caused by functional electrical stimulation. *Journal of Neural Engineering*, 15(2): 026014, January 2018. ISSN 1741-2552. http://dx.doi.org/10.1088/1741-2552/aa9ee8. URL https://doi.org/10.1088%2F1741-2552%2Faa9ee8.

[42] Eric M. Trautmann, Sergey D. Stavisky, Subhaneil Lahiri, Katherine C. Ames, Matthew T. Kaufman, Daniel J. O'Shea, Saurabh Vyas, Xulu Sun, Stephen I. Ryu, Surya Ganguli, and Krishna V. Shenoy. Accurate Estimation of Neural Population Dynamics without Spike Sorting. *Neuron*, 103 (2):292–308.e4, July 2019. ISSN 0896-6273. http://dx.doi.org/10.1016/j.neuron.2019.05.003. URL http://www.sciencedirect.com/science/article/pii/S0896627319304283.

[43] Cynthia A. Chestek, Vikash Gilja, Paul Nuyujukian, Justin D. Foster, Joline M. Fan, Matthew T. Kaufman, Mark M. Churchland, Zuley Rivera-Alvidrez, John P. Cunningham, Stephen I. Ryu, and Krishna V. Shenoy. Long-term stability of neural prosthetic control signals from silicon cortical arrays in rhesus macaque motor cortex. *Journal of Neural Engineering*, 8(4):045005, August 2011. ISSN 1741-2552. http://dx.doi.org/10.1088/1741-2560/8/4/045005. URL http://iopscience.iop.org/1741-2552/8/4/045005.

[44] Breanne P. Christie, Derek M. Tat, Zachary T. Irwin, Vikash Gilja, Paul Nuyujukian, Justin D. Foster, Stephen I. Ryu, Krishna V. Shenoy, David E. Thompson, and Cynthia A. Chestek. Comparison of spike sorting and thresholding of voltage waveforms for intracortical brain–machine interface performance. *Journal of Neural Engineering*, 12(1):016009, December 2014. ISSN 1741-2552. http://dx.doi.org/10.1088/1741-2560/12/1/016009.

[45] D. H. Brainard. The Psychophysics Toolbox. *Spatial Vision*, 10(4):433–436, 1997. ISSN 0169-1015.

[46] David A. Moses, Sean L. Metzger, Jessie R. Liu, Gopala K. Anumanchipalli, Joseph G. Makin, Pengfei F. Sun, Josh Chartier, Maximilian E. Dougherty, Patricia M. Liu, Gary M. Abrams, Adelyn Tu-Chan, Karunesh Ganguly, and Edward F. Chang. Neuroprosthesis for Decoding Speech in a Paralyzed Person with Anarthria. *New England Journal of Medicine*, 385(3): 217–227, July 2021. ISSN 0028-4793. http://dx.doi.org/10.1056/NEJMoa2027540. URL https://doi.org/10.1056/NEJMoa2027540. Publisher: Massachusetts Medical Society _eprint: https://doi.org/10.1056/NEJMoa2027540.

[47] Leo Gao, Stella Biderman, Sid Black, Laurence Golding, Travis Hoppe, Charles Foster, Jason Phang, Horace He, Anish Thite, Noa Nabeshima, Shawn Presser, and Connor Leahy. The Pile: An 800gb dataset of diverse text for language modeling. *arXiv preprint arXiv:2101.00027*, 2020.

[48] J. J. Godfrey, E. C. Holliman, and J. McDaniel. SWITCHBOARD: telephone speech corpus for research and development. pages 517–520. IEEE Computer Society, March 1992. ISBN 978-0-7803-0532-8. http://dx.doi.org/10.1109/ICASSP.1992.225858. URL https://www.computer.org/csdl/proceedings-article/icassp/1992/00225858/12OmNxGSmbC.

[49] Francis R. Willett, Darrel R. Deo, Donald T. Avansino, Paymon Rezaii, Leigh R. Hochberg, Jaimie M. Henderson, and Krishna V. Shenoy. Hand Knob Area of Premotor Cortex Represents the Whole Body in a Compositional Way. *Cell*, March 2020. ISSN 0092-8674. http://dx.doi.org/10.1016/j.cell.2020.02.043. URL http://www.sciencedirect.com/science/article/pii/S0092867420302208.

[50] Shrikanth Narayanan, Asterios Toutios, Vikram Ramanarayanan, Adam Lammert, Jangwon Kim, Sungbok Lee, Krishna Nayak, Yoon-Chul Kim, Yinghua Zhu, Louis Goldstein, Dani Byrd, Erik Bresch, Prasanta Ghosh, Athanasios Katsamanis, and Michael Proctor. Real-time magnetic resonance imaging and electromagnetic articulography database for speech production research (TC). *The Journal of the Acoustical Society of America*, 136(3):1307–1311, September 2014. ISSN 0001-4966. http://dx.doi.org/10.1121/1.4890284. URL https://asa.scitation.org/doi/full/10.1121/1.4890284. Publisher: Acoustical Society of America.

[51] Mark Tiede, Carol Y. Espy-Wilson, Dolly Goldenberg, Vikramjit Mitra, Hosung Nam, and Ganesh Sivaraman. Quantifying kinematic aspects of reduction in a contrasting rate production task. *The Journal of the Acoustical Society of America*, 141(5):3580–3580, May 2017. ISSN 0001-4966. http://dx.doi.org/10.1121/1.4987629. URL https://asa.scitation.org/doi/abs/10.1121/1.4987629. Publisher: Acoustical Society of America.

[52] Daniel Smilkov, Nikhil Thorat, Been Kim, Fernanda Viégas, and Martin Wattenberg. SmoothGrad: removing noise by adding noise, June 2017. URL http://arxiv.org/abs/1706.03825. arXiv:1706.03825 [cs, stat].

[53] Angela Aiello. A Phonetic Examination of California. Master's thesis, UCSC Linguistics Research Center, 2010.

[54] Ludwig Kürzinger, Dominik Winkelbauer, Lujun Li, Tobias Watzel, and Gerhard Rigoll. Ctc-segmentation of large corpora for german end-to-end speech recognition. In *Speech and Computer: 22nd International Conference, SPECOM 2020, St. Petersburg, Russia, October 7–9, 2020, Proceedings 22*, pages 267–278. Springer, 2020.

[55] Alex Graves, Santiago Fernández, Faustino Gomez, and Jürgen Schmidhuber. Connectionist temporal classification: labelling unsegmented sequence data with recurrent neural networks. In *Proceedings of the 23rd international conference on Machine learning*, ICML '06, pages 369–376, Pittsburgh, Pennsylvania, USA, June 2006. Association for Computing Machinery. ISBN 978-1-59593-383-6. http://dx.doi.org/10.1145/1143844.1143891. URL https://doi.org/10.1145/1143844.1143891.

[56] Andrew Senior, Hasim Sak, Felix de Chaumont Quitry, Tara N. Sainath, and Kanishka Rao. Acoustic modelling with cd-ctc-smbr lstm rnns. In *ASRU*, 2015.

[57] Hasim Sak, Andrew W. Senior, Kanishka Rao, Ozan Irsoy, Alex Graves, Françoise Beaufays, and Johan Schalkwyk. Learning acoustic frame labeling for speech recognition with recurrent neural networks. In *ICASSP*, pages 4280–4284, 2015.

[58] Jongseok Park, Kyubyong Kim. g2pe. https://github.com/Kyubyong/g2p, 2019.

[59] Junyoung Chung, Caglar Gulcehre, KyungHyun Cho, and Yoshua Bengio. Empirical Evaluation of Gated Recurrent Neural Networks on Sequence Modeling, December 2014. URL http://arxiv.org/abs/1412.3555. arXiv:1412.3555 [cs].

[60] Beata Jarosiewicz, Anish A. Sarma, Daniel Bacher, Nicolas Y. Masse, John D. Simeral, Brittany Sorice, Erin M. Oakley, Christine Blabe, Chethan Pandarinath, Vikash Gilja, Sydney S. Cash, Emad N. Eskandar, Gerhard Friehs, Jaimie M. Henderson, Krishna V. Shenoy, John P. Donoghue, and Leigh R. Hochberg. Virtual typing by people with tetraplegia using a self-calibrating intracortical brain-computer interface. *Science Translational Medicine*, 7(313):313ra179–313ra179, November 2015. ISSN 1946-6234, 1946-6242. http://dx.doi.org/10.1126/scitranslmed.aac7328. URL http://stm.sciencemag.org/content/7/313/313ra179.

[61] David Sussillo, Paul Nuyujukian, Joline M. Fan, Jonathan C. Kao, Sergey D. Stavisky, Stephen Ryu, and Krishna Shenoy. A recurrent neural network for closed-loop intracortical brain–machine interface decoders. *Journal of Neural Engineering*, 9(2):026027, April 2012. ISSN 1741-2552.

http://dx.doi.org/10.1088/1741-2560/9/2/026027. URL http://iopscience.iop.org/1741-2552/9/2/026027.

[62] Alan D. Degenhart, William E. Bishop, Emily R. Oby, Elizabeth C. Tyler-Kabara, Steven M. Chase, Aaron P. Batista, and Byron M. Yu. Stabilization of a brain–computer interface via the alignment of low-dimensional spaces of neural activity. *Nature Biomedical Engineering*, 4(7): 672–685, July 2020. ISSN 2157-846X. http://dx.doi.org/10.1038/s41551-020-0542-9. URL https://www.nature.com/articles/s41551-020-0542-9. Number: 7 Publisher: Nature Publishing Group.

[63] Daniel Povey, Arnab Ghoshal, Gilles Boulianne, Lukas Burget, Ondrej Glembek, Nagendra Goel, Mirko Hannemann, Petr Motlicek, Yanmin Qian, Petr Schwarz, et al. The kaldi speech recognition toolkit. In *IEEE 2011 workshop on automatic speech recognition and understanding*, number CONF. IEEE Signal Processing Society, 2011.

[64] Mehryar Mohri, Fernando Pereira, and Michael Riley. Speech recognition with weighted finite-state transducers. In *Springer Handbook of Speech Processing*, pages 559–584. Springer, 2008.

[65] Andreas Stolcke, Jing Zheng, Wen Wang, and Victor Abrash. Srilm at sixteen: Update and outlook. In *Proc. IEEE Automatic Speech Recognition and Understanding Workshop*. IEEE SPS, December 2011. URL https://www.microsoft.com/en-us/research/publication/srilm-at-sixteen-update-and-outlook/.

[66] Irving J Good. The population frequencies of species and the estimation of population parameters. *Biometrika*, 40(3-4):237–264, 1953.

[67] Yajie Miao, Mohammad Gowayyed, and Florian Metze. EESEN: End-to-End Speech Recognition Using Deep RNN Models and WFST-Based Decoding. *2015 IEEE Workshop on Automatic Speech Recognition and Understanding (ASRU)*, pages 167–174, 2015.

[68] Zhuoyuan Yao, Di Wu, Xiong Wang, Binbin Zhang, Fan Yu, Chao Yang, Zhendong Peng, Xiaoyu Chen, Lei Xie, and Xin Lei. Wenet: Production oriented streaming and non-streaming end-to-end speech recognition toolkit. In *Proc. Interspeech*, Brno, Czech Republic, 2021. IEEE.

[69] Hy Murveit, John Butzberger, Vassilios Digalakis, and Mitch Weintraub. Large-vocabulary dictation using sri's decipher speech recognition system: Progressive search techniques. In *1993 IEEE International Conference on Acoustics, Speech, and Signal Processing*, volume 2, pages 319–322. IEEE, 1993.

[70] Xavier Aubert and Hermann Ney. Large vocabulary continuous speech recognition using word graphs. In *1995 International Conference on Acoustics, Speech, and Signal Processing*, volume 1, pages 49–52. IEEE, 1995.

[71] Ashish Vaswani, Noam Shazeer, Niki Parmar, Jakob Uszkoreit, Llion Jones, Aidan N Gomez, Łukasz Kaiser, and Illia Polosukhin. Attention is all you need. *Advances in neural information processing systems*, 30, 2017.

[72] Jacob Devlin, Ming-Wei Chang, Kenton Lee, and Kristina Toutanova. Bert: Pre-training of deep bidirectional transformers for language understanding. *arXiv preprint arXiv:1810.04805*, 2018.

[73] Tom Brown, Benjamin Mann, Nick Ryder, Melanie Subbiah, Jared D Kaplan, Prafulla Dhariwal, Arvind Neelakantan, Pranav Shyam, Girish Sastry, Amanda Askell, et al. Language models are few-shot learners. *Advances in neural information processing systems*, 33:1877–1901, 2020.

[74] Susan Zhang, Stephen Roller, Naman Goyal, Mikel Artetxe, Moya Chen, Shuohui Chen, Christopher Dewan, Mona Diab, Xian Li, Xi Victoria Lin, et al. Opt: Open pre-trained transformer language models. *arXiv preprint arXiv:2205.01068*, 2022.

[75] Hasim Sak, Andrew Senior, Kanishka Rao, Ozan Irsoy, Alex Graves, Françoise Beaufays, and Johan Schalkwyk. Learning Acoustic Frame Labeling for Speech Recognition with Recurrent Neural Networks. *2015 IEEE International Conference on Acoustics, Speech and Signal Processing (ICASSP)*, pages 4280–4284, 2015.
