# Supplementary material for: A high-performance speech neuroprosthesis
Source: Nature. 2023 Aug 23;620(7976):1031–6. doi: 10.1038/s41586-023-06377-x (PMC10468393; doi:10.1038/s41586-023-06377-x)
Supplement: Supplementary file 2 — Reporting Summary [file 41586_2023_6377_MOESM2_ESM.pdf]

nature portfolio

Corresponding author(s): Francis R. Willett

Last updated by author(s): Apr 10, 2023

Reporting Summary

Nature Portfolio wishes to improve the reproducibility of the work that we publish. This form provides structure for consistency and transparency in reporting. For further information on Nature Portfolio policies, see our Editorial Policies and the Editorial Policy Checklist.

Statistics

For all statistical analyses, confirm that the following items are present in the figure legend, table legend, main text, or Methods section.

n/a	Confirmed	
☐	☒	The exact sample size (n) for each experimental group/condition, given as a discrete number and unit of measurement
☒	☐	A statement on whether measurements were taken from distinct samples or whether the same sample was measured repeatedly
☐	☒	The statistical test(s) used AND whether they are one- or two-sided<br>*Only common tests should be described solely by name; describe more complex techniques in the Methods section.*
☒	☐	A description of all covariates tested
☐	☒	A description of any assumptions or corrections, such as tests of normality and adjustment for multiple comparisons
☐	☒	A full description of the statistical parameters including central tendency (e.g. means) or other basic estimates (e.g. regression coefficient) AND variation (e.g. standard deviation) or associated estimates of uncertainty (e.g. confidence intervals)
☐	☒	For null hypothesis testing, the test statistic (e.g. F, t, r) with confidence intervals, effect sizes, degrees of freedom and P value noted<br>*Give P values as exact values whenever suitable.*
☒	☐	For Bayesian analysis, information on the choice of priors and Markov chain Monte Carlo settings
☒	☐	For hierarchical and complex designs, identification of the appropriate level for tests and full reporting of outcomes
☐	☒	Estimates of effect sizes (e.g. Cohen's d, Pearson's r), indicating how they were calculated

Our web collection on statistics for biologists contains articles on many of the points above.

Software and code

Policy information about availability of computer code

Data collection	The software for running th experimental tasks, recording data and real-time sentence decoding was a custom developed system using MATLAB, Simulink Real-Time, and Python. Software packages used included tensorflow 2.10.0, gp2_en 2.1.0, WeNet, SRILM and Kaldi.
Data analysis	Data was analyzed using custom MATLAB and Python code. Code is publicly available on GitHub here: https://github.com/fwillett/speechBCI

For manuscripts utilizing custom algorithms or software that are central to the research but not yet described in published literature, software must be made available to editors and reviewers. We strongly encourage code deposition in a community repository (e.g. GitHub). See the Nature Portfolio guidelines for submitting code & software for further information.

Data

Policy information about availability of data

All manuscripts must include a data availability statement. This statement should provide the following information, where applicable:

- Accession codes, unique identifiers, or web links for publicly available datasets
- A description of any restrictions on data availability
- For clinical datasets or third party data, please ensure that the statement adheres to our policy

All neural data needed to reproduce the findings in this study are publicly available on Dryad here: (link & DOI to be added - under review at Data Dryad now). The dataset contains neural activity recorded during the attempted speaking of 10,850 sentences, as well as instructed delay experiments designed to investigate the neural representation of orofacial movement and speech production. As part of this study, we also analyzed publicly available electromagnetic articulography data:

the USC-TIMIT database (https://sail.usc.edu/span/usc-timit/) and the Haskins Production Rate Comparison (HRPC) database (https://yale.app.box.com/s/cfn8hj2puveo65fq54rp1ml2mk7moj3h).

Research involving human participants, their data, or biological material

Policy information about studies with human participants or human data. See also policy information about sex, gender (identity/presentation), and sexual orientation and race, ethnicity and racism.

Reporting on sex and gender	This study included data from one participant, T12, who is a biological female and identifies as a woman. This information was self-reported. No sex or gender based analyses were performed given there was only a single participant and the study was assessing brain-computer interface performance.
Reporting on race, ethnicity, or other socially relevant groupings	This study assessed brain-computer interface performance for a single participant. No variables relating to race, ethnicity or other socially relevant groupings were reported or analyzed.
Population characteristics	This study includes data from one participant (identified as T12) who gave informed consent and was enrolled in the BrainGate2 Neural Interface System clinical trial (ClinclaTrials.gov Identifier: NCT00912041, registered June 3, 2009) but this study did not report clinical trial results. T12 is a left-handed woman, 67 years old during data collection with bulbar ALS that began approximately 9 years prior to enrollment.
Recruitment	Participant T12 was enrolled in the BrainGate 2 clinical trial after meeting inclusion criteria based in part on disease characteristics. Inclusion and exclusion criteria are available online (ClinicalTrials.gov).
Ethics oversight	The BrainGate2 Neural Interface System clinical trial was approved under an Investigational Device Exemption (IDE) by the US Food and Drug Administration (IDE #G09003). Permission was also granted by teh Institutional Review Board of Stanford University (protocol #20804). All research was performed in accordance with relevant guidelines/ regulations.

Note that full information on the approval of the study protocol must also be provided in the manuscript.

Field-specific reporting

Please select the one below that is the best fit for your research. If you are not sure, read the appropriate sections before making your selection.

☒ Life sciences ☐ Behavioural & social sciences ☐ Ecological, evolutionary & environmental sciences

For a reference copy of the document with all sections, see nature.com/documents/nr-reporting-summary-flat.pdf

Life sciences study design

All studies must disclose on these points even when the disclosure is negative.

Sample size	No sample-size calculation was performed. Data were collected in a single participant to characterize the performance of a brain-computer interface. Uncertainty in performance estimates were quantified with confidence intervals, and show a robust result.
Data exclusions	This study is based on brain-computer interface performance evaluation data collected over a series of days. All days are reported in the study and all relevant data is included.
Replication	This study assessed brain-computer interface performance with a single participant. Results were replicated across eight independent days of performance evaluation.
Randomization	Randomization into groups is not relevant for this study as only one participant is included in the study.
Blinding	Blinding is not relevant to this study as only one participant was included to asses the performance of a brain-computer interface.

Reporting for specific materials, systems and methods

We require information from authors about some types of materials, experimental systems and methods used in many studies. Here, indicate whether each material, system or method listed is relevant to your study. If you are not sure if a list item applies to your research, read the appropriate section before selecting a response.

Materials & experimental systems

n/a	Involved in the study
☒	☐ Antibodies
☒	☐ Eukaryotic cell lines
☒	☐ Palaeontology and archaeology
☒	☐ Animals and other organisms
☒	☐ Clinical data
☒	☐ Dual use research of concern
☒	☐ Plants

Methods

n/a	Involved in the study
☒	☐ ChIP-seq
☒	☐ Flow cytometry
☒	☐ MRI-based neuroimaging
